# The novel resveratrol analogue HS-1793 induces apoptosis via the mitochondrial pathway in murine breast cancer cells

**DOI:** 10.3892/ijo.2012.1615

**Published:** 2012-08-31

**Authors:** HYOUN-JI KIM, KWANG-MO YANG, YOO-SOO PARK, YOO-JIN CHOI, JI-HYEON YUN, CHEOL-HUN SON, HONG-SUK SUH, MIN-HO JEONG, WOL-SOON JO

**Affiliations:** 1 Department of Research Center, Dong Nam Institute of Radiological and Medical Sciences, Busan 619-953;; 2 Department of Biochemistry, College of Medicine, Pusan National University, Busan 609-735;; 3 Department of Microbiology, College of Medicine, Dong-A University, Busan 602-714, Republic of Korea

**Keywords:** resveratrol analog, HS-1793, apoptosis, mitochondria, caspase, breast cancer

## Abstract

Resveratrol (3,4′,5 tri-hydroxystilbene), a natural plant polyphenol, has gained interest as a non-toxic chemopreventive agent capable of inducing tumor cell death in a variety of cancer types. Several studies were undertaken to obtain synthetic analogues of resveratrol with potent anticancer activity. The aim of the present study was to investigate the effect of HS-1793 as a new resveratrol analog on apoptosis via the mitochondrial pathway in murine breast cancer cells. A pharmacological dose (1.3–20 μM) of HS-1793 exerted a cytotoxic effect on murine breast cancer cells resulting in apoptosis. HS-1793-mediated cytotoxicity in FM3A cells by several apoptotic events including mitochondrial cytochrome *c* release, activation of caspase-3 and PARP occurred. In addition, HS-1793 induced collapse of ΔΨ_m_ and enhanced AIF and Endo G release from mitochondria while undergoing apoptosis. These results demonstrate that the cytotoxicity by HS-1793 in FM3A cells can mainly be attributed to apoptosis via a mitochondrial pathway by caspase activation or contributions of AIF and Endo G.

## Introduction

Although disease-free survival and overall survival of patients with breast cancer have been improved through intensive treatment, breast cancer is still an important public health problem in women worldwide (1,2). Major breast cancer treatment methods consist, both separately and in combination of surgery, radiotherapy and chemotherapy. In particular, tamoxifen, anti-estrogen medicine, is widely used in the prevention and treatment of estrogen receptor positive breast cancer (3). Inherent or acquired tumor drug resistance limits many agents that could be used to treat this disease and are often associated with severe, dose-limiting and systemic toxicities. Therefore, new agents acting on novel targets in breast cancer are currently under investigation and the need to develop novel non-toxic therapeutic agents active against breast cancer remains an important goal.

Interest in the pharmacological effects of bioactive compounds on cancer treatments and prevention has increased dramatically over the past twenty years. A great number of natural agents derived from plants can be potentially useful in complementary therapy for cancer patients. As well, there is a need to develop a new, more powerfully active drug from natural resources with lesser side effects that can act as a substitute for the current chemical therapy. A recent study confirmed that increasing vegetable and fruit consumption might reduce the risk of breast cancer (4,5). Also a lower incidence of breast cancer is associated with a high consumption of phytoestrogens, which are biologically active plant-derived phenolic compounds that structurally mimic the mammalian estrogen, estradiol-17b (6,7). Among many bioactive compounds, basic and preclinical research on resveratrol (*trans*-3,4′,5-trihydroxystilbene), a naturally occurring polyphenol which can be found in grapes and red wine, has shown pleiotropic, cardioprotective, anti-aging and anticancer activities (8,9). In addition, resveratrol is not a potent cytotoxic compound when compared with other chemotherapeutic drugs. However, exposure to high doses of resveratrol is required to induce apoptosis in cancer cells and resveratrol’s biological activity is limited by its photosensitivity and metabolic instability. Thus, several studies were undertaken to obtain synthetic analogues of resveratrol with potent anti-cancer activity (10). We previously investigated, designed and synthesized analogues of resveratrol that had potent activity and demonstrated that four synthetic resveratrol analogues (HS-1784, -1792, -1791 and -1793) displayed stronger antitumor effects than resveratrol in most cancer cells tested, including human breast adenocarcinoma cell line MCF-7 (11–13). Moreover, the resveratrol analogue, 4-(6-hydroxy-2-naphthyl)-1,3-benzenediol (HS-1793), especially overcomes the resistance conferred by Bcl-2 by inducing apoptosis (11,13).

Taken together, the evidence from *in vitro* studies indicated that the anticancer activity of HS-1793 in various cancer cells was mediated through apoptosis (11,13). However, the specific apoptosis mechanisms at work are not yet well understood. Therefore, the aim of the present study was to investigate whether HS-1793 induced cytotoxicity via mitochondria induced apoptosis and explore the potential mechanisms in murine breast cancer cells.

## Materials and methods

### Chemical and reagents

RPMI-1640 medium and fetal bovine serum (FBS) were obtained from Gibco (Gaithersburg, MD, USA). JC-1 was obtained from Molecular Probes (Eugene, OR, USA). Rabbit monoclonal to cytochrome *c*, PARP [poly(ADP-ribose) polymerase], caspase-3 and GAPDH antibodies were obtained from Abcam (Cambridge, UK). Anti-AIF (apoptosis-inducing factor) and anti-Endo G (endonuclease G) rabbit antibodies were obtained from Calbiochem (San Diego, USA). Propidium iodide (PI) was obtained from Sigma (St. Louis, MO, USA). The enhanced chemiluminescent western blotting detection reagent (SuperSignal West Pico chemiluminescent substrate) was obtained from Pierce (Rockford, IL, USA).

### Preparation of HS-1793

To obtain HS-1793, the stilbene double bond present in resveratrol was substituted with a naphthalene ring as previously described (11,12). A stock solution was made in absolute ethanol at 50 mM, and the working dilutions were directly made in culture media. The control vehicle was culture media containing amounts of ethanol equivalent to those present in HS-1793.

### Cell culture condition

FM3A (murine breast cancer cells) originated from the mammary gland of the C3H/He mouse were grown in RPMI-1640 medium (Gibco) containing 10% FBS, 100 U/ml penicillin and 100 U/ml streptomycin, under the condition of wet air containing 5% CO_2_ and 37°C.

### HS-1793 treatment and cell viability assay

HS-1793 dissolved in EtOH (50 mM) was prepared and stored at −80°C until use. FM3A cells were plated onto 6-well plates at a density of 2×10^5^ cells/well and grown for treatment period. For dose-response experiments, different concentrations of HS-1793 (0, 1.3, 2.5, 5, 10, 15 and 20 μM) were added to cells and grown at 37°C and 5% CO_2_ for 24 and 48 h. For time-dependent experiments, the cells were treated with 5 μM of HS-1793 for 0, 12, 24, 36, 48 and 72 h. Cell viability determined with hemacytometer by trypan blue and MTT assay as previously described (11).

### Sub-G1 phase assay

FM3A cells were precultured onto 6-well plates at a density of 2×10^5^ cells/well for 24 h and the cells were treated with HS-1793 (5 μM) according to exposure time (0, 12, 24, 36, 48 and 72 h). The trypsin was added to the cells in plates for 3 min, after which the cells were harvested by centrifugation at 1,500 rpm for 5 min. The pellets were washed twice with cold phosphate-buffered saline (PBS) and then fixed by using 70% ethanol at 4°C for 30 min. The cells were then washed twice with cold PBS and resuspended in PBS containing 40 μg/ml PI soultion, 0.1 mg/ml RNase and 0.1% Triton X-100 in a dark room for 30 min at 37°C. The cells were then analyzed by flow cytometer (Beckman Coulter, USA). The sub-G1 (apoptosis) phase analyzed with software for cell cycle analysis.

### Nuclear morphology analysis of apoptosis

FM3A cells were pretreated with HS-1793 for each exposure time (0, 12, 24, 36, 48 and 72 h). Then, cells were harvested and cell suspension was centrifuged onto a clean, fat-free glass slide with a cytocentrifuge. The samples were fixed for 10 min in 4% paraformaldehyde (PFA) and stained with 4 μg/ml of Hoechst 33342 at 37°C for 30 min. The cells were photographed under Nikon Eclipse TS100 fluorescence microscopy (Nikon, Tokyo, Japan).

### TUNEL assay

Apoptosis was detected by using TUNEL apoptosis detection kit (Millipore, USA) and performed according to the instruction of the supplier. The samples were observed under fluorescence microscopy.

### Assay of mitochondrial membrane potential (ΔΨm)

Change in ΔΨ_m_ were determined by staining the cells with the potential-sensitive fluorescent probe 5,5′,6,6′-tetrachloro-1,1′3,3′-tetraethylbenzimidazol carbocyanine iodide JC-1. The JC-1 dye was directly added to 1 μM of final concentration into the cell culture medium. After incubation for 20 min, the cells were centrifuged at 1,000 rpm for 5 min and removed supernatant. Then, the cells were washed with medium and added to 400 μl of PBS. ΔΨ_m_ was measured by a flow cytometer.

### Confocal laser microscopy

FM3A cells were pretreated with HS-1793 for each exposure time (0, 12, 24, 36, 48 and 72 h). The cells were incubated for 15 min at 37°C after adding 450 μM of mitotracker probe (Invitrogen, Carlsbad, CA, USA) and then washed with medium. The cells were cytospan onto a clean fat-free glass slide. Cytocentrifuged cells were fixed in 4% PFA for 30 min and incubated overnight at 4°C with anti-cytochrome *c*, AIF and Endo G antibody (1:100 dilution) and then incubated with secondary antibody (FITC-conjugated goat anti-rabbit IgG at 1:100 dilution) for 1 h at 37°C. Photomicrographs were obtained using a Zeiss LSM 700 laser-scanning confocal microscope (Zeiss, Goettingen, Germany).

### Immunoblotting

The cell were harvested and washed twice with ice-cold PBS. Then, cells were resuspended in 200 μl ice-cold solubilizing buffer (300 mM NaCl, 50 mM Tris-HCl, pH 7.6, 0.5% Triton X-100, 1 ml protease inhibitor cocktail) and incubated at 4°C for 40 min. The lysates were centrifuged at 14,000 rpm for 20 min. Protein concentrations of cell lysates were determined by using the Bio-Rad protein assay kit (Bio-Rad Laboratories, Hercules, CA, USA). Equal amounts of protein were subjected to 7.5–15% SDS-PAGE for caspase-3, PARP, anti-cleaved caspase-3 and anti-cleaved PARP, respectively, and transferred to a nitro-cellulose membrane. Western blot analysis was performed using standard protocols (14). Immunostaining with antibodies was performed using Super-Signal West Pico enhanced chemiluminescence substrate and detected with LAS-3000PLUS (Fuji Photo Film Co., Kanagawa, Japan).

### Statistical analysis

All data are expressed as mean standard deviation (SD). The evaluation of statistical significance was performed using Student’s t-test or one-way analysis of variance (ANOVA) using the Statistical Package for the Social Sciences (SPSS) statistical software for Windows, Version 18.0 (Chicago, IL, USA). For all analyses, a difference was considered to be significant at P<0.05.

## Results

### HS-1793 reduced cell viability in FM3A cells

**Figure 1.**
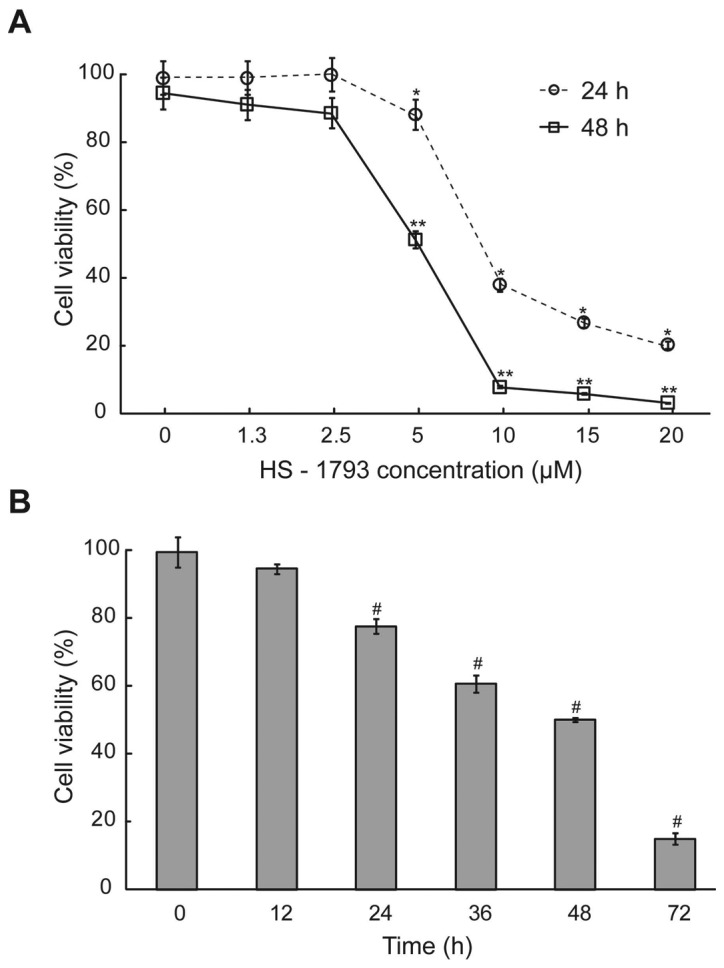
The effect of cell viability after HS-1793 treatment in FM3A cells. (A) The cells were treated with or without different concentrations (0, 1.3, 2.5, 5, 10, 15 and 20 μM) of HS-1793 for 24 or 48 h. (B) After treatment of HS-1793 5 μM, the cells were treated for various exposure times (0, 12, 24, 36, 48 and 72 h). Cells were collected by centrifugation and the viable cells were counted by trypan blue or MTT assay. Data are reported as the mean ± SD of three experiments. ^*,**,#^P<0.05 as compared with untreated control (0 μM).

To examine the antitumor activity of HS-1793 in murine breast cancer cells, exponentially dividing FM3A cells were treated with various concentrations of HS-1793, and cell viability was measured at each exposure time. HS-1793 significantly decreased the percentages of viable cells and those effects were dose-dependent at 24 and 48 h (p<0.05, Fig. 1A). However, HS-1793 induced no significant inhibition of cell growth in a nonmetastatic human mammary epithelial cell line (MCF-10A) under tested concentrations (data not shown). Indeed, HS-1793 caused marked growth inhibition of 50% (IC_50_) in FM3A cells at 5 μM for 48 h and we also showed that HS-1793 treatment significantly inhibited FM3A cell growth in a time-dependent manner at 5 μM (p<0.05, Fig. 1B). Therefore, our data indicate that HS-1793 induced effective cell death in FM3A cells in a dose- and time-dependent manner.

### HS-1793 increased sub-G1 phase and induction of apoptosis in FM3A cells

**Figure 2.**
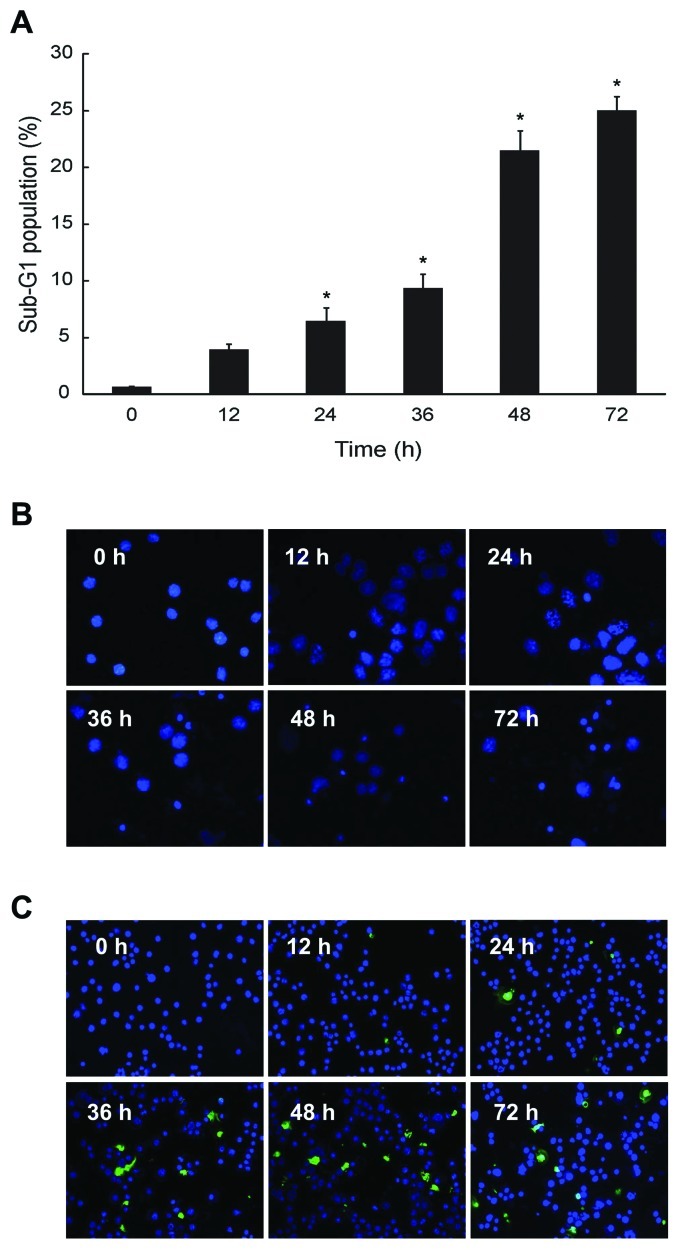
The effects of HS-1793 on apoptosis in FM3A cells. The cells were incubated with 5 μM of HS-1793 for various exposure times (0, 12, 24, 36, 48 and 72 h). (A) The cells were collected by centrifugation and sub-G1 phase was analyzed by flow cytometry. Data are reported as the mean ± SD of three experiments. ^*^P<0.05 as compared with untreated control (0 μM). The cells were photographed by (B) Hoechst 33342 staining and (C) TUNEL assay under a fluorescence microscope (×200).

We investigated whether the HS-1793 induced cytotoxicity in FM3A cells would be due in part to proapoptotic effects. Consequently, the effect of HS-1793 on cell cycle progression was analyzed by flow cytometry in exponentially dividing cultures of FM3A cells treated with either ethanol (control) or HS-1793 (5 μM), and the percentages of cells in sub-G1 phases were calculated. HS-1793 induced enhancement of the sub-G1 DNA content in a time-dependent manner at 5 μM (Fig. 2A). In addition, we identified apoptotic features in the cells using nuclear morphological changes and DNA fragmentation. FM3A cells exhibited increase of nuclear fragment (Fig. 2B) and DNA fragment (Fig. 2C) in a time-dependent manner by HS-1793 treatment (5 μM). However, HS-1793 did not induced an enhancement of sub-G1 DNA content, nuclear fragment and DNA fragment at 5 μM in MCF-10A (data not shown). These results suggest that HS-1793 can have anti-proliferative activity through induction of apoptosis in FM3A cells while there is little normal cell toxicity under treatment condition.

### HS-1793 changed ΔΨ_m_ levels in FM3A cells

**Figure 3.**
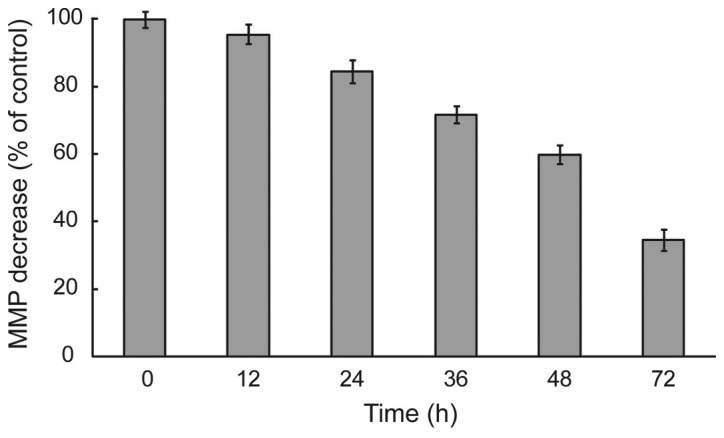
The effect of HS-1793 on mitochondria membrane potential (ΔΨ_m_) levels in FM3A cells. The cells were incubated with 5 μM of HS-1793 for various exposure times (0, 12, 24, 36, 48 and 72 h) and the percentage of cells that were stained individually JC-1 probe for ΔΨ_m_. The stained cells were examined and quantitated by flow cytometry. Data are reported as the mean ± SD of three experiments. ^*^P<0.05 as compared with untreated control (0 μM).

During apoptosis, early and pivotal events occurred in the mitochondria that were often, although not always, associated with the collapse in ΔΨ_m_(14). To delineate this mechanism, we measured whether HS-1793 induced alterations of ΔΨ_m_ by the use of mitochondrial selective lipophilic cation JC-1 probe. HS-1793 exerted enhancement of JC-1 green fluorescence intensity after treatment of HS-1793 (5 μM), representative cells with depolarized mitochondria in a time-dependent and significant depolarization with 2.9-fold decrease in ΔΨ_m_ were observed after treatment at 72 h in FM3A cells (p<0.05, Fig. 3). It can be seen in that HS-1793 decreased the levels of ΔΨ_m_ in FM3A cells and these effects were time-dependent.

### HS-1793 promotes release of cytochrome c, AIF and Endo G from the mitochondria into the cytosol

**Figure 4.**
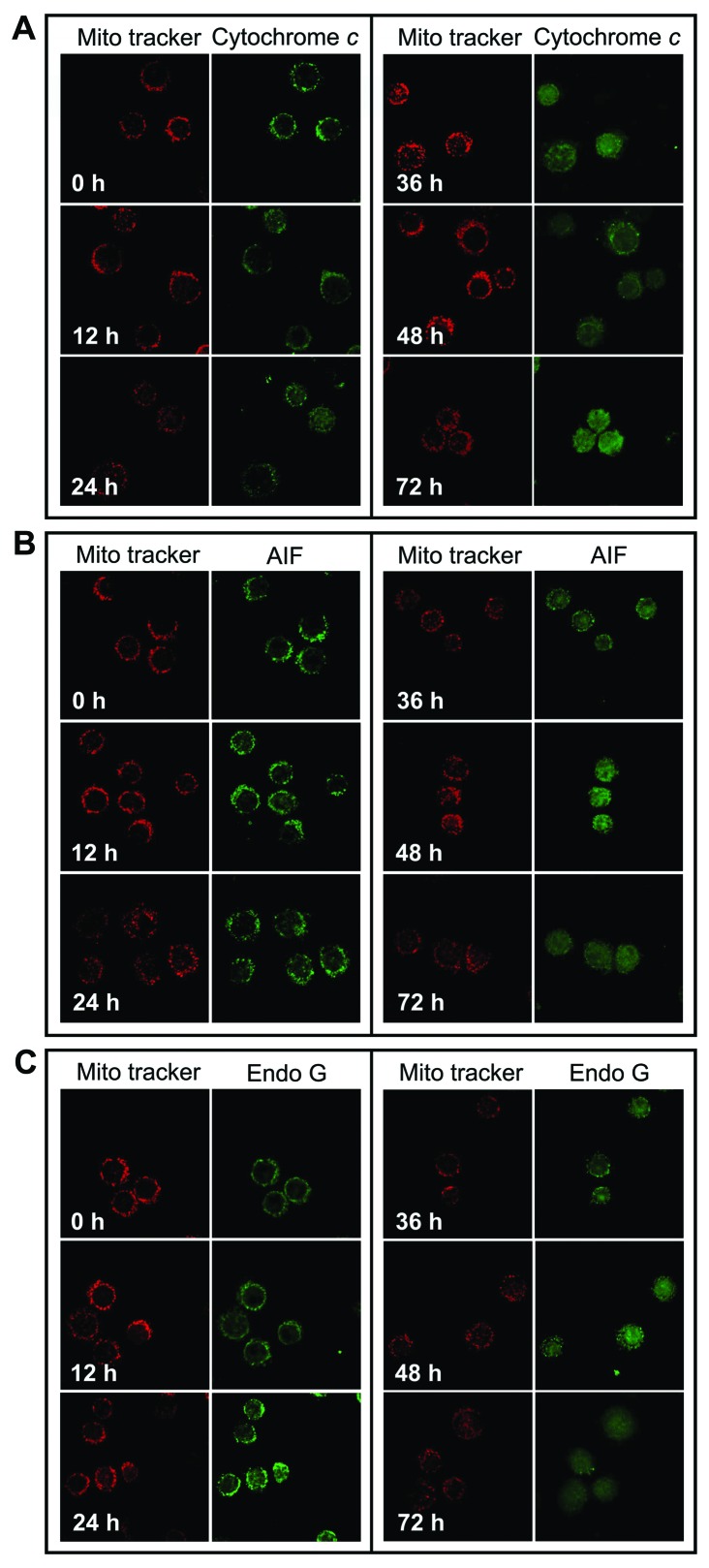
The effect of HS-1793 on cytochrome *c*, AIF and Endo G translocations from mitochondria in FM3A cells. The cells were incubated with 5 μM of HS-1793 for various exposure times (0, 12, 24, 36, 48 and 72 h). The cells were fixed and stained with primary antibodies to (A) cytochrome *c*-, (B) AIF- (B) and (C) Endo G-labeled secondary antibodies were used (green fluorescence) and labeled with mito tracker dye for mitochondria. The proteins were detected by a confocal laser microscopic system (×400).

We investigated whether cytochrome *c*, Endo G and AIF were released from the inner mitochondrial membrane during HS-1793 treatment. FM3A cells after treatment with 5 μM of HS-1793 for 12, 24, 36, 48 and 72 h, cells were harvested and the translocation of cytochrome *c*, AIF and Endo G were determined using confocal laser microscopy. Fig. 4 shows that HS-1793 promotes the release of cytochrome *c* (Fig. 4A), AIF (Fig. 4B) and Endo G (Fig. 4C) from mitochondria and that longer treatment time periods increased the release of the mitochondrial proteins.

### HS-1793 induces apoptosis by caspase-3 and PARP activation in FM3A cells

**Figure 5.**
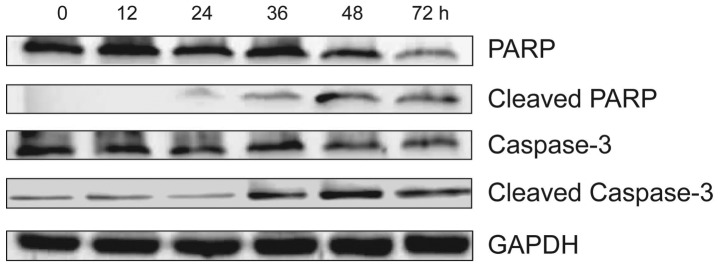
The effect of HS-1793 on the expression of apoptosis-related proteins in FM3A cells. The cells were incubated with 5 μM of HS-1793 for various time periods (0, 12, 24, 36, 48 and 72 h) and the total proteins were collected and quantitated. Subsequently, caspase-3 and PARP were measured by western blotting.

In order to confirm that HS-1793 induced apoptosis was also mediated through mitochondria-dependent caspase activation, we analyzed caspase-3 and PARP activities by using western blotting and treatment with 5 μM of HS-1793 in FM3A cells for different time-points (0–72 h). As shown in Fig. 5, HS-1793 decreased caspase-3 and PARP expression in FM3A cells while cleaved caspase-3 and PARP expression was increased by HS-1793. These data suggest that HS-1793 induces activation of caspase-dependent pathway in FM3A cells, therefore the caspase cascade would be involved in the apoptosis.

## Discussion

The therapeutic goal for cancer is to trigger tumor-selective cell death and the tumor’s response to therapy depends mainly on tumor’s ability to undergo cell death. The role of apoptosis in the cytotoxicity of anticancer drugs has become clearer (15). Among natural bioactive compounds, the antiproliferative activity of the resveratrol against tumor cell lines of different origins has been extensively characterized (16–23). From these studies, it was found that resveratrol induced cell death and that, in certain cell types, it involved an apoptotic mechanism (18,21–23). The dose at which an apoptotic effect was seen in resveratrol is relatively higher (100–200 μM) than the dose used to induce cell cycle arrest or cancer cell proliferation inhibition (10–30 μM) (20,22). *In vitro* studies demonstrated that resveratrol exerts dose- and time-dependent antiproliferative and proapoptotic effects in human breast cancer MCF-7 and MDAMB-231 cells, thus decreasing cell viability (22). A key target for identifying methods of cancer prevention and therapy is the induction of apoptosis or the debilitation of cancer cells without excessive normal cell damage by any natural compound (24,25). In this respect, chemical modification of the stilbene backbone of resveratrol may need to enhance its biological activity. Previous studies have reported that several resveratrol analogues demonstrate stronger anti-tumor effects than resveratrol (10,11). Among them, HS-1793 does not contain the unstable double bond found in resveratrol and the position of two of three hydroxyl groups in HS-1793 at the aromatic ring is different from resveratrol (12). The term resveratrol derivative/analogue is used for HS-1784 because HS-1784 is a derivative of resveratrol and HS-1793 is derived from HS-1784 which has been reported in previous studies (11,12). A synthetic analogue having the same structure as HS-1784 was documented to have a high ceramide-mediated proapoptotic activity in human breast cancer cells and to block the cell cycle in the G0–G1 phase in leukemia cells (26). HS-1793 was also noted to display stronger antitumor effects than resveratrol in most cancer cells, to overcome the resistance conferred by Bcl-2 in U937 cells via 14-3-3, and to exert its antitumor activity via Bad (11). However, there is still considerable uncertainty about the cytotoxic effects on HS-1793 induced apoptosis mechanism in breast cancer cells.

In search for novel strategies for further management of breast cancer, we have attempted to identify the molecular mechanisms involved in HS-1793-induced apoptosis, both caspase-dependent and -independent via mitochondria pathway. In the present study, we found that HS-1793 was effective in decreasing cell numbers in the murine FM3A breast cancer cell line through growth inhibition and/or apoptosis. Furthermore, to understand the association between HS-1793 and apoptosis, we showed various apoptotic changes in FM3A cells exposed to 5 μM of HS-1793. In sub-G1 DNA content, HS-1793 was enhanced in a time-dependent manner and also increased nuclear fragment and DNA fragment. Our results showed that HS-1793 induced apoptosis or cell growth inhibition in lower dose (3–25 μM) than resveratrol (100–300 μM) in breast cancer cells (11,22). These results suggest that HS-1793, a novel resveratrol analogue, may be superior to natural resveratrol as a candidate for chemoprevention agent.

Most of the conventional anticancer treatments are thought to induce cell death through indirect activation of the mitochondria-dependent pathway of apoptosis, a pathway often found altered in drug-resistant cancer cells (27,28). In most cases, chemotherapeutic drugs first interact with an intracellular target resulting in stress signals that secondarily converge to mitochondria and finally result in apoptotic cell death. Following stress signals generated by conventional treatments, the permeability of mitochondrial membranes is increased, leading to the release of proapoptotic proteins which in turn initiate the caspase cascade and finally result in cell death (27,29). In particular, a mitochondria-dependent step, involving outermembrane permeabilization, is associated with most pro-apoptotic stimuli. The mitochondria contain several apoptogenic factors (cytochrome *c*, Smac/Diablo, HtrA2/Omi, AIF and Endo G) and the release of these factors regulate apoptosis (30). Endo G and AIF have been reported to induce caspase-independent nuclear apoptosis and thus it has been proposed that they are involved in caspase-independent cell death processes (31–35). AIF is a phylogenetically ancient mitochondrial intermembrane flavoprotein endowed with the unique capacity to induce caspase-independent peripheral chromatin condensation and large-scale DNA fragmentation and provides a biochemical link between the associated mitochondrial membrane permeabilization and the nuclear signs of apoptosis (31,35). Endo G is a mitochondrial nuclease that is likely implicated in mitochondrial DNA replication and is synthesized as a propeptide with an amino-terminal presequence that targets the nuclease to mitochondria (34). Loss of ΔΨ_m_ induced secretion of apoptotic proteins such as AIF and Endo G proteins from mitochondria to cytosol promoted the activation of apoptosis. AIF and Endo G cleave DNA in the nucleus leads to caspase-independent cell death (28,36). In this study, we investigated whether the mitochondrial AIF and Endo G release involving ΔΨ_m_ were critical for the HS-1793-mediated apoptosis. Our data clearly indicated that HS-1793 caused loss of ΔΨ_m_ the translocations of cytochrome *c*, AIF and Endo G protein from the nucleus into cytosol at 5 μM concentration in a time-dependent manner in FM3A cells. It can be seen that HS-1793 promoted the release of AIF and Endo G from mitochondria into cytosol and enhanced caspase-independent death in FM3A cells.

The loss of membrane potential is an early event in mitochondrial-mediated apoptosis (36,37). After the reduction of membrane potential and the release of mitochondrial cytochrome *c*, a critical step is the formation of apoptosomes, which ultimately cleave procaspase-3 to form active caspase-3. Caspases play critical roles in the execution of apoptosis (38,39). Caspase-3 has also been shown to be a key component integral for apoptosis, and relies on the actions of the initiator caspases including caspase-8 and -9 to mediate its actions (40). In addition, molecular studies examining the apoptotic process have revealed that the Bcl-2 protein functions upstream of caspase-3, and that it prevents the proteolytic activation of caspase-3, thus leading to cleavage of PARP and apoptosis (40–43). Our results showed that HS-1793 treatment activated caspase-3 as well as PARP in FM3A cells. HS-1793 induced cytotoxicity through increases of DNA fragmentation, nuclear fragmentation and sub-G1 DNA contents as well as the cleavage of PARP, thus confirming that the apoptosis induced by HS-1793 in FM3A cells might be mediated through the caspase-3 pathway.

In conclusion, these results demonstrate that HS-1793 induced cytotoxicity in FM3A cells is due to subsequent induction of apoptosis via mitochondrial pathway from caspase activation or cytochrome *c*, AIF and Endo G release. These findings suggest that HS-1793 might be a potentially promising candidate compound that needs to be further explored as an anticancer agent or mitochondria target drugs for the treatment of human breast cancer.
